# Oxidative stress-induced telomeric erosion as a mechanism underlying airborne particulate matter-related cardiovascular disease

**DOI:** 10.1186/1743-8977-9-21

**Published:** 2012-06-19

**Authors:** Thomas J Grahame, Richard B Schlesinger

**Affiliations:** 1 United States Department of Energy, 1000 Independence Avenue, SW Washington, DC, 20585, USA; 2 Department of Biology and Health Sciences, Dyson College of Arts and Sciences Pace University, One Pace Plaza, New York, NY, USA

**Keywords:** Telomere, Oxidative stress, Particulate matter, Cardiovascular disease, Black carbon, Vehicular emissions

## Abstract

Particulate matter (PM) pollution is responsible for hundreds of thousands of deaths worldwide, the majority due to cardiovascular disease (CVD). While many potential pathophysiological mechanisms have been proposed, there is not yet a consensus as to which are most important in causing pollution-related morbidity/mortality. Nor is there consensus regarding which specific types of PM are most likely to affect public health in this regard. One toxicological mechanism linking exposure to airborne PM with CVD outcomes is oxidative stress, a contributor to the development of CVD risk factors including atherosclerosis. Recent work suggests that accelerated shortening of telomeres and, thus, early senescence of cells may be an important pathway by which oxidative stress may accelerate biological aging and the resultant development of age-related morbidity. This pathway may explain a significant proportion of PM-related adverse health outcomes, since shortened telomeres accelerate the progression of many diseases. There is limited but consistent evidence that vehicular emissions produce oxidative stress in humans. Given that oxidative stress is associated with accelerated erosion of telomeres, and that shortened telomeres are linked with acceleration of biological ageing and greater incidence of various age-related pathology, including CVD, it is hypothesized that associations noted between certain pollution types and sources and oxidative stress may reflect a mechanism by which these pollutants result in CVD-related morbidity and mortality, namely accelerated aging via enhanced erosion of telomeres. This paper reviews the literature providing links among oxidative stress, accelerated erosion of telomeres, CVD, and specific sources and types of air pollutants. If certain PM species/sources might be responsible for adverse health outcomes via the proposed mechanism, perhaps the pathway to reducing mortality/morbidity from PM would become clearer. Not only would pollution reduction imperatives be more focused, but interventions which could reduce oxidative stress would become all the more important.

## Introduction

Epidemiological studies have found associations between exposure to airborne particulate matter (PM) and adverse effects on the cardiovascular system, as well as increased incidence or prevalence of cardiovascular disease (CVD)-related morbidity and mortality [1,2]. However, the pathophysiology underlying this relationship has not been clearly defined. One mechanism underlying PM-related CVD is oxidative stress. This occurs when the homeostatic balance of oxidizing agents to anti-oxidants is upset towards an imbalance of the former. The primary oxidizing agents in this regard are reactive oxygen species (ROS), such as hydroxyl radical and superoxide anion. While these are normal products of aerobic metabolism, under conditions whereby ROS production increases beyond the ability to detoxify these chemicals, molecular damage may result since ROS readily reacts with proteins, lipids and nucleic acids.

An American Heart Association Scientific Statement on Particulate Matter has proposed a role for oxidative stress in altering cardiac function [2]. A review of the relationship between oxidative stress and air pollutants discussed the role of fine PM, and certain more biologically active components thereof, in causing both primary oxidizing effects in lung lining fluid and a secondary wave of oxidative stress via influx of inflammatory cells [3]. A recent report from the Health Effects Institute hypothesized that oxidative stress might indeed be the underlying mechanism of action by which exposure to pollutants specifically from traffic may lead to adverse health outcomes [4]. Furthermore, a review of *in vitroin vivo* and epidemiological studies examining vehicular-derived PM emissions and their association with cardiovascular morbidity and mortality concluded that one of the major toxicological mechanisms likely linked to CVD is oxidative stress [5]. Finally, there is increasing evidence that some specific components of airborne PM that may contribute little to total PM_2.5_ mass may be responsible for inducing oxidative stress [6].

Advanced age is a known risk factor for a number of chronic diseases and functional impairment, including those involving the cardiovascular system. However, as opposed solely to chronological age, “biological” age, which is determined both by physiology as well as chronology, appears to be a key factor related to development of ultimate pathology and this, in turn, has been related to a cumulative burden of oxidative stress [7]. There is also increasing evidence that biological aging is related to shortening of chromosomal telomeres.

Telomeres are structures consisting of noncoding nucleotide sequences (telomeric DNA), associated with certain proteins, found at both ends of chromosomal DNA molecules. They help protect against erosion of coding DNA, which would lead to subsequent loss of genetic information on the chromosomes during repetitive replication cycles.

Several proteins associated with telomeric DNA, including telomerase, a reverse transcriptase enzyme, and the telomeric repeat binding factors 1 and 2 (TRF1, TRF2), help regulate telomere length and structure. Telomerase is involved in the replication of telomeric DNA strands, so as to preserve their length when chromosomal DNA is replicated. However, in most mammalian somatic cells, as opposed to germ and stem cell lines, activity of telomerase is very low, or even absent [8,9], resulting in incomplete replication of the telomere and the loss of a certain number of telomeric DNA base pairs during each division cycle with subsequent shortening of the telomere itself. Because progressive shortening of telomeres is a crucial factor in the normal process of organismal aging, telomeric length can serve as a marker of a cell’s biological age, in contrast to actual chronological age [10,11].

The natural erosion of telomeric length is cumulative. Eventually, with repetitive cell divisions, the telomere reaches a critical length, and further cell division can no longer proceed. The cell then becomes senescent, and eventually undergoes apoptosis. Senescent cells have been reported to accumulate in tissues and organs, with potential to interfere with normal tissue function and structure. Senescence has also been causally related to the generation of age-related phenotypes, since removal of senescent cells seems to prevent or delay tissue dysfunction [12].

It is of interest to note that significant telomerase activity has been found in approximately 90% of human cancers, regardless of cell type [13,14], in contrast to normal somatic cells as previously noted. The abnormally increased levels of telomerase allow these cells to continue to divide rather than become senescent with repeated DNA replication cycles [9]. However, when telomerase activity in these cells was inhibited, telomeres became shorter with each division, and eventually cell growth ceased.

The rate of telomeric erosion over time is determined partially by genetic factors and partially by environmental factors, and their interaction likely modulates biological aging, affecting the risk of developing age-related diseases [15]. A role for biological age “advancement” in heart failure and the pathogenesis of myocardial infarction (MI) has been suggested [16], and shortened telomeres appear to be responsible for accelerating several important CVD disease states and risk factors, including hypertension and atherosclerosis [17].

This paper reviews the relationship between telomeric length, CVD, and oxidative stress, and discusses a role for specific PM air pollutants and pollutant sources in inducing oxidative stress. Based upon the available evidence, we propose that a potential mechanism underlying at least some PM exposure association with CVD involves an accelerated rate of cell telomere erosion due to oxidative stress, resulting in an acceleration of the rate of biological aging. This may be an important and generalized mechanism underlying PM-related increases in CVD morbidity and mortality.

### Linking oxidative stress and telomere length

The ability of oxidative stress to damage DNA provides a potential mechanism by which it could interfere with replication of telomeric DNA, accelerating the rate of telomeric erosion. A study examining the effect on circulating leukocyte telomeric length in people in relation to status of the UCP2 gene, which is involved in the regulation of mitochondrial ROS production, found that the presence of a functional promoter UCP2 variant that resulted in increased oxidative stress via down regulation of gene expression was associated with shorter telomeric length than when the variant was absent, suggesting a direct link between ROS and accelerated telomeric erosion [18]. Telomere DNA is very prone to such oxidative damage since it is especially rich in guanine; accumulation of this damage along the telomere during the lifespan of a cell can then result in enhanced telomere loss during each replication cycle, thus contributing to accelerated cell senescence [19,20].

Reactive oxygen species can damage DNA either directly or during attempted repair of oxidation-induced base modifications [20]. In addition, as already noted, telomeric DNA is inefficient in repair of single strand breaks or other induced lesions, in contrast to most genomic DNA, in that repair is either slower or more incomplete [21]. This inefficiency may be further enhanced by oxidative damage to any telomerase which may be present in the cell. For example, increased levels of intracellular ROS have been shown to result in a reduction of telomerase activity, which can be prevented by the presence of ROS scavengers which also delay the onset of cellular senescence [22]. Thus, due to the sensitivity of both telomerase and telomeric DNA to oxidative-induced damage and the normal insufficiency of repair mechanisms, telomeres are especially sensitive to cumulative oxidative damage [23]. Therefore, oxidative stress is a likely environmental factor accelerating telomeric erosion at each replication cycle, with resultant accelerated cellular biological aging [24].

The extent of telomeric erosion has been related to the level of oxidative stress. Richter and van Zglinicki [25] examined the rate of telomeric shortening under conditions of oxidative stress induced by varying levels of oxygen in cultures of fibroblasts obtained from human adult skin, foreskin, embryonic lung and sheep embryos. The investigators reported a correlation between ROS levels and the rate of telomeric shortening, which was independent of the individual human donor or specific animal species. They further suggested that a given level of intracellular ROS would lead to a predictable acceleration of the rate of shortening of telomeres over the norm, regardless of any other difference between donors in terms of individual genetics within one species, or between species. Furthermore, high levels of intracellular ROS and accumulated damage to DNA and protein have been associated with the development of senescent somatic cells, compared with much less oxidative damage noted in more “immortal” cell types that can continue to divide for longer periods of time [26,27].

If oxidative stress plays a role in telomeric erosion, then the presence of antioxidants should result in some modulation of this effect. Overexpression of extracellular superoxide dismutase resulted in a decrease in both the peroxide content of human fibroblasts, and the rate of telomeric shortening [28]. An association between oxidative stress and increased rate of telomeric shortening, likely due to direct ROS-induced damage to telomeric DNA, and the preventative role of antioxidants in decelerating this rate, has been shown in a number of other *in vitro* studies as well [19,21,29,30].

The role of oxidative stress as an environmental factor modulating telomeric length has also been suggested by epidemiological studies. Most of these involved assessment of CVD, and are discussed in subsequent sections. Acceleration of the appearance of age-related pathology is supported by a study that found leukocyte telomere length associated with age-related disease burdens across multiple physiological systems, and that was independent of its association with actual chronological age [31]. This provided direct evidence that mechanisms which shorten telomere length, such as oxidative stress, will likely cause progression of a variety of diseases, including CVD.

### Linking CVD and telomere length

A number of epidemiological studies involving diverse populations have consistently show an association between shorter telomeric length in cells obtained from patients with CVD compared to healthy people. One such study examined the relationship between telomeric length in circulating leukocytes and various indices or risk factors for clinical CVD in a group of 419 adults aged over 65 yrs (mean age 74.2 yr) [32]. It noted a statistically significant to borderline significant inverse relationship between telomere length and C-reactive protein, IL-6, carotid artery intima media thickness, and ankle brachial index, but only in those aged 73 yrs or younger. It appears that the effect of shorter telomere length on disease outcome attenuates above a certain chronological age. In another study, leukocyte mean telomere length was examined in a group of people having mean age of 78 yr, and then in surviving members of this group seven years later and having mean age of 83 yr [33]; shorter length was predictive of CVD-related mortality only for men under 80 yrs of age. On the other hand, some studies have found no association between telomere length and carotid artery media thickness [34,35]. The reasons for the apparent inconsistency are unclear, but may be a function of the specific populations examined in this regard.

Another study compared leukocyte telomere length in a population of 484 men (age 45-65 yr) who subsequently developed coronary heart disease with that from 1058 matched controls who did not have any CVD events [36]. Those in the mid and lowest tertiles of telomeric length at the time of recruitment into the study had approximately a two fold greater risk of developing some form of coronary heart disease over the next 4.9 yr than did those in the highest tertile. This study, similar to the study noted above [33], strongly suggested that mean leukocyte telomere length was a predictor of future CVD, and that the relationship between telomeric length and CVD was not a consequence of the disease but, rather, a factor in its development.

Similarly, a study of 143 people over the age of 60 yrs found that the presence of shorter than population average telomere length was associated with a greater than threefold increase in cardiac mortality over approximately the following 9 years [37]. An association of reduced telomere length with increased risk of myocardial infarction (MI) and other cardiac diseases, and with greater mortality rate, has also been found [38]; the risk of MI, considered as premature MI, in people under age 50 was higher for those with shorter telomere lengths compared to those with lengths in the highest quartile. Attrition of telomere length has also been noted in people showing age-related cardiomyopathy [39]. Telomere shortening has also been linked to CVD via impairment of cardiac stem cell-mediated myocardial regeneration, and may also contribute to CVD by inducing myocardial cell apoptosis apart from any effect on stem cells [40,41].

A potential familial predisposition to CVD, perhaps related to telomeric length, was described by Wong et al. [42]. They measured leukocyte telomeric length in patients having ischemic heart failure and in their high risk children, and noted that length was shorter in cells from patients and their children compared to healthy controls and their children. A study using biopsy material [39] found that myocytes from hearts of diseased elderly people showed a 39% reduction in average telomeric length, and a higher degree of necrosis and apoptosis, than did comparable cells from healthy controls. Another study [41] noted that telomeric length in leukocytes of people with dilated, failing hearts, compared with those from aged-matched healthy controls, had lengths that were 25% shorter, decreased expression of TRF-2, and marked activation of a kinase involved in DNA repair.

Most studies of telomere length *in vivo* used circulating leukocytes as they are easy to obtain. Kuznetsova et al. [43] addressed the issue of whether telomeric length in circulating leukocytes was representative of that in the cardiac myocytes themselves. It appears that the rate of telomeric shortening is indeed similar in different tissues of any individual and, therefore, easily accessible cells such as circulating leukocytes can indeed serve as surrogates for telomere length assessment in those cells involved in specific disease processes, such as vessel endothelium or cardiac myocytes [20,44,45].

Since shortening of telomeric length is associated with cell senescence and biological age, and with diseases normally associated with increasing chronological age, one of the ways in which differences in telomeric length in people with or without CVD may be expressed is by the number of years of chronological age to which this difference in length can be equated [7]. In most subjects under the age of 70 yrs, there seems to be a consistent chronological to biological age difference equivalent of between 8 and 12 yrs [36,38]. In other words, those people who will go on to develop or already have CVD show telomeres with average lengths equivalent to healthy people who are chronologically 8 to 12 yrs older.

Specific risk factors for CVD or specific CVD-related conditions have also been examined in relation to telomere length. Telomere length in leukocytes was found to be related to altered left ventricular mass, a determinant of cardiac function, both longitudinally and cross-sectionally, in a study involving 334 people with mean age of 46.5 yr [43]. Shorter mean telomere length in leukocytes has also been reported to be associated with a decreased left ventricular ejection fraction in the elderly who had no prior evidence of cardiac disease [46]. Chronic heart failure, a condition in which the heart has lost the ability to pump enough blood to the body's tissues, is often the result of valvular heart disease, such as aortic valve stenosis, that results in ventricular hypertrophy and resulting compromised cardiac function. Decreased leukocyte telomere length has been found to be associated with degenerative aortic stenosis [47].

Increased cardiomyocyte apoptosis is a characteristic of chronic heart failure [41,48]. Telomerase knockout mice, in which telomeres show increased rate of shortening, have attenuated myocyte division, increased rate of apoptosis, and cardiomyocyte hypertrophy, all leading to cardiac remodeling that mimics human endstage dilated cardiomyopathy [49]. This suggests that telomeres may play a primary role in CVD through their impact on cellular senescence, and that shorter telomere length associated with CVD may not be merely a reflection of the greater extent of vascular cell turnover that occurs in these conditions [7]. On the other hand, the increased cell turnover noted could result in even further enhanced telomere attrition than would occur normally [50]. Telomerase activity has also been found to be significantly reduced in patients (median age 80 – 82 yr) with congestive heart failure compared to healthy controls [51].

The extent of coronary atherosclerosis, indicated by the occurrence and relative extent of coronary artery calcification, may be a predictor of a coronary event and an index of the biological age of the artery [52-55]. A cross-sectional study examined the relationship between telomere length and calcification in a group of 325 adults (aged 40-64 yr) [56]. An inverse relationship was noted between telomere length and coronary arterial calcification in people with no clinical history of cardiac disease. Shorter mean telomere length in leukocytes has also been reported to be associated with an increased tendency of carotid artery atherosclerosis in hypertensive individuals [57].

The relationship between arterial tissue and telomere length was examined in 84 subjects (mean age 69 yr). Telomere length was shorter in aortic tissue which showed atherosclerosis than did corresponding tissue without these lesions [58]. Another study [59] examined telomere length in leukocytes and atherosclerotic plaques from patients with carotid atherosclerosis and in blood cells of age and gender-matched controls who had no clinical evidence of atherosclerosis. Telomere length was shorter in patients than in controls, although length in the plaque cells was only weakly correlated with that in the white cells.

## Linking CVD, oxidative stress and telomere length

The discussion in the previous sections provided evidence that telomere length is modulated by oxidative stress, and that shortened telomere length may be a factor in the pathogenesis of CVD. This section integrates these concepts, discussing the relationship between oxidative stress, telomere length, and overt CVD or risk factors.

A known risk factor for CVD is chronic psychological stress [60,61], the proposed mechanism for which is psychological stress-induced oxidative stress due to autonomic nervous system and neuroendocrine responses [11]. For example, adrenal glucocorticoid hormones have been shown to increase oxidative stress-related neuronal damage [62,63].

Epel et al. [11] studied leukocyte telomere length in a group of 50 women, 20-50 yrs of age (mean 38yr), who were undergoing chronic or perceived life stress but were otherwise healthy. They found both perceived and chronic stress to be positively correlated with oxidative stress, using F2–isoprostane in urine, a biomarker of lipid peroxidation. They postulated that psychological stress can induce early cell senescence leading to CVD. In another study, leukocyte telomere length was examined in a small group of people with chronic depression [64]. Various markers of oxidative stress were assessed; telomere length was inversely correlated with the level of oxidative stress in all subjects.

As noted above, telomere length can reflect biological aging and the risk of CVD. Telomere length in endothelial progenitor cells from patients with CVD was found to be shorter, and telomerase activity lower, than in healthy matched controls; oxidative stress in the ill subjects was also greater [65]. In a longitudinal study involving a cohort of 2059 people aged 35-55 yr (average 46 yrs) who were free of any overt CVD [50], baseline leukocyte telomere length showed no significant correlation with single measures of cholesterol HDL level and blood pressure, known CVD risk factors, but shorter length was significantly associated with increased levels of several markers of oxidative stress. This study suggested that telomere length reflected the burden of oxidative stress seemingly independent of life style, as indicated by these other classical risk factors. However, the rate of leukocyte telomere shortening has been associated with longitudinal “cumulative” levels of cholesterol HDL [66] suggesting, as noted above, that telomere length may be reflective of an accumulating lifetime burden of oxidative stress, and that more “instant” markers of risk taken at any one time may not be as reflective and, therefore, may show no clear association with length.

There is other evidence for the relation between CVD and oxidative stress. For example, lipid peroxidation induced by ROS is an early event in the atherosclerotic process [67]. Hydrogen peroxide and oxidized low density lipoproteins have been found to inhibit endothelial cell telomerase activity [68]. Furthermore, long-term exposure of human vascular endothelial cells to oxidative stress induced by alteration of the glutathione redox cycle resulted in down-regulation of telomerase activity, enhanced erosion of telomeres, and early onset of cellular senescence [69].

Diabetes, another risk factor for CVD, is associated with high levels of oxidative stress resulting in cellular damage and accelerated cellular aging [16]. Impairment in the function of cardiac progenitor cells involved in regeneration of damage in the cardiovascular system resulting from diabetes-enhanced oxidative stress is one of the likely underlying causes of diabetic cardiomyopathy. Type 2 diabetes has also been associated with shorter telomeric length in white blood cells, and diabetes appears to accelerate telomeric shortening in these cells in patients having a history of MI.

In sum, the available evidence supports a role for oxidative stress in accelerating telomere shortening, inasmuch as diseases in which chronic oxidative stress is a hallmark are associated with shortened length. This, in turn, provides support for an association between accelerated rate of telomere erosion due to oxidative stress and CVD pathophysiology. Furthermore, differences in telomere length in individuals with or without CVD cannot be consistently explained merely by differences in some classical risk factors for CVD other than age, such as obesity, BMI, smoking status, or cholesterol level [32,36,38,50,70]. At best, there is only little to moderate association with shorter lengths and these factors, if any relationship is noted at all, suggesting that oxidative stress is a potentially greater risk factor. Thus, a reduction in the cellular replicative capacity of cardiac or other tissues within the cardiovascular system due to oxidative stress-induced accelerated erosion of telomere length may be directly involved in the progression to CVD, and may even determine its severity [43].

## Linking exposure to PM_2.5_ species or sources and telomere length or oxidative stress

### Linking with telomere length

There is some, albeit limited, evidence that exposure to traffic-related emissions can modulate telomere length. In a study conducted in Milan, Italy [71], mean telomere length was found to be significantly shorter in traffic officers than in office worker controls, and in traffic officers working in high traffic vs. those working in low traffic areas. Telomere length also decreased with increasing chronological age in both traffic officers and office workers, but was shorter in traffic officers for each age category and was also decreased with increased exposure levels to vehicular emissions (i.e., benzene and toluene).

In a study of 165 veterans in the greater Boston area, McCracken et al. [17] modeled black carbon (BC) concentrations in the homes of subjects, and found that an interquartile increase of BC (0.25 μg/m^3^) was associated with a significant decrease in telomere length; statins, which have both anti-inflammatory and antioxidant properties, had a protective effect on telomere shortening.

### Linking with oxidative stress

This section reviews human panel studies which link traffic-related emissions to several different measures of oxidative stress. Some studies compared oxidative stress in people exposed to traffic or diesel emissions for several hours or during a workshift, compared to matched controls not so exposed, while others linked levels of specific PM_2.5_ species with oxidative stress. Because oxidative stress is also associated with telomere shortening, as reviewed in previous sections, these studies would then provide a link to traffic-related emissions generally, or specific components of such emissions.

Studies which involved only measures of total PM mass have not been included herein, because they cannot inform us as to which specific PM_2.5_ species are associated with health risks. On the other hand, we have emphasized studies which: (A) assessed either at least two specific PM_2.5_ species or total PM_2.5_ mass and at least one PM_2.5_ species including BC/EC; and (B) have accurate exposure information for those chemical species exhibiting local variability. Regarding (A), it is possible that a significant association for a pollutant variable might exist only because of the lack of measurement of another important PM_2.5_ species. Regarding (B), recent studies [72,73] have found that accurate subject exposure accounted for the difference between finding small, insignificant health risk associations when a central monitor was used to characterize overall exposure for everyone within a large geographic area and greater, more significant, associations using a more accurate exposure metric, e.g., monitors outside or inside residences, or personal monitors. A summary of relevant human panel studies meeting the criteria noted above is provided in Table 1. Table 2 illustrates the differences in findings in one study [72] when exposure to a specific pollutant, namely black carbon, was assessed using personal monitors compared to when exposure assessment utilized a central monitor.

**Table 1:** **Human panel studies with good subject exposure methodologies associating measures of oxidative stress with different pm**_**2.5**_**species or specific sources**

***Study***	***Oxidative stress measures***	***Subjects***	***PM***_***2.5***_***species, other measures of exposure***	***Associations found***	***Accuracy of subject exposure to BC/EC***^**1**^
Delfino et al., 2008 [74]	Circulating GPx-1, Cu, Zn-SOD	29 non-smoking elderly w/CAD in Los Angeles area, many on statins or anti-hypertension meds; 12 weekly blood draws	Ambient PM_0.25_, PM_2.5_, PN, EC, OC, BC, OC_pri_, SOA	For Cu, Zn-SOD: Associations found with PM_0.25_, PM_2.5_, EC, BC, OC_pri_, but not with OC, PN, SOA	Very good: monitors inside and outside retirement communities
Delfino et al., 2009 [75]	Circulating GPx-1, Cu, Zn-SOD	60 non-smoking elderly w/CAD in Los Angeles area, many on statins or anti-hypertension meds; 5-12 weekly blood draws	Ambient PM_0.25_, PM_2.5_, PN, EC, OC, OC_pri_, SOA	For Cu, Zn-SOD: Associations (stronger in cool season) with PM_0.25_, PM_2.5_, EC, PN, OC_pri_, but not with OC, SOA	Very good: monitors inside and outside retirement communities
Kim et al., 2004 [76]	8-OHdG	20 boilermakers, average 45.5 year of age, repairing oil-fired boilers in Boston area	Industrial levels of PM_2.5_, PM_2.5_ metals V, Cr, Mn, Ni, Cu, Pb (PM_2.5_ = 440 μg/m^3^, V = 1.23 μg/m^3^, other metals < 1.0 μg/m^3^)	Post work shift 8-OHdG levels significantly higher than pre shift; increased PM_2.5,_ V, Mn, Ni, Pb concentrations significantly associated with increased 8-OHdG	Excellent: personal monitors used
Loft et al., 1999 [77]	8-oxodG	57 non-smoking diesel bus drivers in greater Copenhagen	Ambient air in urban (30 drivers) vs. rural/suburban (27 drivers) areas	Significantly higher 8-OHdG excretion in urban drivers vs. rural/suburban drivers	Very good: exposure is based upon comparison of urban (heavily trafficked) vs. rural/suburban areas
Sauvain et al., 2011 [78]	8-OHdG	32 Swiss bus maintenance workers	PM_4_, OC, EC, PM metal (Fe, Mn, Cu) content, PAHs (PM_4_ between 25 and 71 μg/m^3^)	8-OHdG excretions significantly increased within each shift and between two consecutive work days; increases in non-smokers associated with increases in OC and particulate Cu	Very good: associations based upon exposure measured indoors, as nearby as possible to work stations
Wei et al., 2009 [79]	8-OHdG	2 non-smoking young security guards working by a major road in Beijing, noon to 8 PM; worksite ambient PM_2.5_ = 243 μg/m^3^, background PM_2.5_ = 104 μg/m^3^	Pre and post-shift 8-OHdG samples collected for 29 days; associations with four “clusters” of PM_2.5_species (PM_2.5_ mass, PAHs, metals, polar organic species)	Post work shift increases in 8-OHdG significantly associated with PM_2.5_ mass, PAHs, and metals, but not with polar organic species, as measured at worksite	Very good exposure : taken at worksite
Lee et al., 2011 [80]	8-OHdG	28 diesel exhaust inspectors in Taiwan, 38 age and gender matched controls office workers, monitored during 3 consecutive day work periods	Diesel PM_2.5_ emissions (DEP_2.5_), PAH content of DEP_2.5_ (personal daily PM_2.5_ = 86 to 94 μg/m^3^, PAHs = 3.04 to 4.11 ng/m^3^)	8-OHdG levels significantly higher for inspectors vs. controls on days 2 and 3; increased PAH concentrations in DEP_2.5_ significantly associated with increased 8-OHdG in exposed group, after adjusting for smoking status and BMI	Very good: personal monitors and ambient samplers installed within meters of work sites
Lai et al., 2004 [81]	8-OHdG	47 female highway toll workers in Taiwan, 27 female office workers as controls	Exposed (toll workers, 8 hour shifts) vs. office workers	Toll workers had significantly higher 8-OHdG levels than controls (86% higher 8-OHdG comparing non-smokers to non-smokers); increases in 8-OHdG almost 5 times higher per 1000 trucks/buses than per 1,000 cars, result not significant (separate lanes for different vehicle types)	Very good: exposure defined by whether working in traffic or not; for those working in traffic, further defined by car lane or truck/bus lane workers and by traffic density
Sorensen et al., 2005 [82]	8-oxodG	49 non-smoking students in Copenhagen, median age 24, in each of two seasons (summer, autumn)	PM_2.5_, six metals in PM_2.5_ fraction (V, Cr, Pt, Ni, Cu, Fe); PM_2.5_ = 20.7 μg/m^3^ (fall), 12.6 μg/m^3^ (summer)	No associations found with urinary 8-oxodG; significant positive associations found for V, Cr with 8-oxodG in lymphocytes. 98 samples available for urinary 8-oxodG, 52 for lymphocytes	Excellent; weekly personal monitors concentrations for each season
Allen et al., 2009 [83]	8-OHdG, F2-isoprostanes	10 adults, ages 18-49, with metabolic syndrome but no history of ongoing medical care for heart disease, hypertension, asthma, diabetes, or other chronic condition	2 hour exposure to either diesel exhaust (200 PM_2.5_) from engine operating at 75% of rated output, or filtered air	No significant increase in 8-OHdG or F2-isoprostanes levels after exposure, in double blind crossover experiment	Excellent: exposure to diesel emissions or filtered air occurred in chamber designed for purpose
Jacobs et al., 2011 [84]	Oxidized LDL	79 non-smoking diabetics, average age 56.5 years, in urban Belgium	Carbon area in airway macrophages, distance of residences from major roads	Increase in IQR carbon loading, decrease in distance from major roads both associated with increase in oxidized LDL	Excellent: carbon loading in personal macrophages is a precise measure of particulate carbon, which in urban area is from traffic, mainly diesels
Kipen et al., 2011 [85]	White blood cell (WBC), red blood cell (RBC) proteasome activity,	38 healthy young subjects in New Jersey	Diesel exhaust (DE), laboratory-generated secondary organic aerosol (SOA, based upon gas phase reactions of ozone and d-limonene), both ~ 190 μg/m^3^	Compared to filtered air exposure, significant decreases of WBC proteasome activity after exposure to either DE or SOA, decrease of RBC proteasome activity after exposure to DE, “presumably via induction of oxidative stress”	Excellent: exposure chamber used
Adar et al., 2007 [86]	HRV measures: SDNN, r-MSSD, PNN50 + 1, LF, HF, LF/HF, HR	44 non-smoking subjects over age 60 living in 4 seniors’ residences in St. Louis	BC, PM_2.5_ levels as recorded by mobile monitor which followed subjects, including in traffic on bus	For 24 average BC concentrations, for 5 minute concentrations on diesel bus or not on bus, and for 5 and 24 hour means, the great majority of many BC associations are significant; PM_2.5_ highly correlated with BC and associations similar to those for BC	Very good (monitor follows subjects during day, stays in residence of subjects at night)
Schwartz et al., 2005 [87]	4 measures of HRV	27 subjects living adjacent to major urban road in Boston, age 61-89	BC, PM_2.5_, “secondary PM” derived by subtracting BC mass from PM_2.5_ mass	BC: 7 of 8 associations significant	Very good (monitor adjacent to same major urban road to which subjects’ residences also adjacent)
				PM_2.5_: 3 of 8 associations significant	
				“Secondary PM”: no significant associations of 8	
Ebelt et al., 2005 [88]	SDNN, R-MSSD measures of HRV	16 non-smoking patients with COPD in Vancouver, average age = 74	PM_2.5_, sulfate, estimated non-sulfate PM_2.5_	Significant associations only with non-sulfate PM_2.5_	Very good (personal monitoring information combined with ambient monitoring information, to separate effects)
				For SDNN, 2 of 2 tests	
				For R-MSSD, 1 of 2 tests	
Creason et al., 2001 [89]	HF, LF measures of HRV	56 elderly, non-smoking men, residents of a retirement center near Baltimore, near commuter roads	PM_2.5_ (sulfate monitored but not used in models; expressed as percentage of PM_2.5_ in discussion)	For complete dataset of 24 days, insignificant associations with either HF or LF, with a “U” shaped function where HRV measures are at normal levels only at lowest and highest PM_2.5_ concentrations; when a two day period with no effects on HRV is removed, remaining 22 days have a significant, linear reduction in both HF and LF, for both indoor and outdoor PM_2.5_; two days removed had highest and 3^rd^ highest PM_2.5_ levels, were high in sulfate but came from rural areas with no apparent urban or industrial source of harmful PM_2.5_	Reasonably good in that study was able to determine by wind back-trajectory analysis that for 22 days where PM_2.5_ reduced HRV measures, air parcels passed over either urban or industrial areas, but that for the 2 days without effect on HRV, parcels passed over more rural areas
Suh and Zanobetti, 2010 [72]	4 measures of HRV: SDNN, RMSSD, PNN50, HF, LF/HF	Same as in Wheeler et al. (2006)	EC, sulfate, PM_2.5_	IQR increase in *personal* monitored EC significantly associated with decreases in SDNN, RMSSD, PNN50, and HF, and with increase in LF/HF; IQR increase in *ambient* monitored EC not associated with changes in any HRV measures; Neither sulfate nor PM_2.5_ (only personal monitored available for sulfate) significantly associated with any HRV measures	Excellent for personal monitored EC, PM_2.5_, and sulfate; Poor (horizontal exposure misclassification; central monitor reading for people across metro Atlanta area) for ambient EC
Park et al. (2007) [90]	SDNN, HF, LF, LF/HF	497 subjects of Normative Aging Study living across greater Boston area	BC, sulfate, PM_2.5_, combined with wind trajectory information showing source directions	Four of six trajectories had high and nearly equal concentrations of BC, sulfate, and PM_2.5_ - associations are for these four trajectories.	Poor for BC (horizontal exposure misclassification; central monitor reading for people across metro Boston area); however, use of wind trajectory analysis mitigates poor exposure by allowing interpretation of rural vs. urban sources
				For local (stagnant) wind trajectory: BC (2 significant tests of 4); Sulfate (1 of 4); PM_2.5_ (1 of 4)	
				For long distance urban wind trajectory from southwest (over major urban areas): BC (3 of 4 tests significant);	
				For two non-urban trajectories: BC, sulfate, PM_2.5_ (none significant)	
Langrish et al., 2009 [91]	SDNN, LF	15 healthy, non-smoking young volunteers in Beijing	Personal monitor PM_2.5_, randomized cross over study design: volunteers walked a predetermined city center route with or without a face mask	24 hour SDNN significantly lower when face mask not used; LF significantly lower (one test) without mask, but interpretation not straightforward	Excellent; most important finding is corroboration that urban particulate matter, not gases, drives reduction in SDNN, suggesting associations in other studies with BC/EC indicate direct PM effects, BC/EC not a proxy for vehicular gaseous emissions
Langrish et al., 2012 [92]	HF, RMSSD	98 patients with coronary artery disease, average age 62	Personal monitor PM_2.5_, randomized cross over study design: volunteers walked a predetermined city center route with or without a face mask	HF, RMSSD significantly lower when face mask not used	Excellent; most important finding is corroboration that urban particulate matter, not gases, drives reduction in RMSSD, HF, suggesting associations in other studies with BC/EC indicate direct PM effects, BC/EC not a proxy for vehicular gaseous emissions

Abbreviations: GPx-1 = glutathione peroxidase-1; Cu, Zn-SOD = copper-zinc superoxide dismutase; CAD = coronary artery disease; PN = particle number; EC = PM_2.5_ elemental carbon; BC = PM_2.5_ black carbon; OC = PM_2.5_ organic carbon; SOA = estimated secondary organic carbon; OC_pri_ = primary OC; NAS = Normative Aging Study, a cohort of male veterans across greater Boston area, many of whom are present or former smokers, and many of whom are on anti-hypertensive medications or statins; 8-OHdG or 8-oxodG = 8-hydroxy-2’-deoxyguanosine, a product of oxidation of the deoxynucleotide pool and a repair product of oxidation of guanine in DNA (mutagenic); IQR = interquartile range; PAH = polycyclic aromatic hydrocarbon; BMI = body mass index, a generally reliable indicator of body fat; HRV = Heart rate variability, of which there are several measures; SDNN = standard deviation of consecutive RR Intervals (an HRV measure); r-MSSD or RMSSD = root-mean square of the difference of successive R-R intervals (an HRV measure); PNN50 + 1 = number of instances per hour in which two consecutive R-R intervals differ by more than 50 ms over 24 h (an HRV measure); HF = high frequency component of HRV; LF = low frequency component of HRV; LF/HF = ratio of LF to HF; TP = Total power (an HRV measure); COPD = Chronic Obstructive Pulmonary Disease; MI = Myocardial Infarction; IQR = Interquartile Range of pollutant.

^1^Accuracy of subject exposure is considered very good to excellent if assessment of concentration of pollutant and actual subject exposure vary well together with time because measurement is taken in close proximity to subjects. Accuracy is seem as “horizontally poor” if a central monitor is used to characterized subject exposure over a large geographical area, and is additionally “vertically poor” if the monitor is several hundred feet higher than the residences of the subjects.

**Table 2:** **Association between 24-hour ambient monitor and personal EC concentrations with different hrv measures (from Suh and Zanobetti**[72]**)**

***HRV measure***	**Ambient (Central monitor)**		**Personal monitor**	
	***Change (%)***	***95% confidence interval***	***Change (%)***	***95% confidence interval***
SDNN	-1.0	-3.7 to 1.7	-**4.66**	-**7.99 to** -**1.22**
RMSSD	-3.6	-9.5 to 2.8	-**10.97**	-**18.00 to** -**3.34**
PNN50	-0.34	-10.55 to 11.04	-**15.16**	-**26.33 to** -**2.29**
HF	-2.36	-11.67 to 7.92	-**13.41**	-**23.95 to** -**1.41**
LF/HF ratio	2.60	-1.89 to 7.29	**6.22**	**0.15 to 12.64**

Results in boldface indicate statistical significance at the 95% confidence level.

#### Studies providing direct measures of oxidative stress

As noted previously, oxidative stress in this paper has been defined as an imbalance between the generation and removal of free radicals. Air pollution studies have used several direct markers of this oxidative stress response. These include the following:

· circulating biomarkers of erythrocyte antioxidant activity in blood, such as glutathione peroxidase-1 (GPx-1) and copper-zinc superoxide dismutase (Cu, Zn-SOD).

· 8-hydroxy-2’-deoxyguanosine (designated as either 8-OHdG or 8-oxodG) in urine or in lymphocytes, a product of oxidation of the deoxynucleotide pool, and of oxidation of guanine in DNA.

· increases in redox-sensitive transcription factors.

· differences in oxidative stress-related gene activity among subjects exposed to pollutants.

Delfino et al. [74,75] examined elderly non-smoking retirees having coronary artery disease and living in several centers across the Los Angeles area. Pollution monitors were located both inside and outside residences, and measures of oxidative stress included circulating biomarkers in blood (GPx-1, Cu, Zn-SOD). These are among the few studies that considered secondary organic aerosols (SOA), which in Los Angeles would mainly be oxidation products of carbonaceous gases of vehicular origin, among the different PM_2.5_ species being considered. The studies found associations for Cu, Zn-SOD with primary PM_2.5_ species mainly of vehicular origin (EC, BC, primary organic carbon, PM_0.25_), but not SOA or mixed secondary and primary organic species (OC). Inorganic secondary aerosols, such as sulfate, were not monitored, presumably because major sulfate sources, such as coal-fired power plants, were several hundred miles downwind from the study area.

Loft et al. [77] examined 8-OHdG levels in 57 bus drivers in Copenhagen, 30 who drove urban routes and 27 who drove rural/suburban routes. 8-OHdG excretion was significantly elevated in the former compared to the latter. Although urban atmospheres appeared to be the cause of oxidative stress, this study did not assess specific vehicular-derived emissions.

Wei et al. [79] examined pre- and post-work shift 8-OHdG levels in security workers in Beijing, whose work site was on a heavily trafficked road. Four “clusters” of PM species were examined: PM_2.5_ mass, PAHs, metals, and polar organic species. Observed post-shift increases in urinary 8-OHdG were associated with increased levels of PM_2.5_ mass, PAHs, and metals, but not polar organic species, at the work site.

Lee et al. [80] studied 28 diesel exhaust inspectors in Taiwan, age and gender matched with office worker controls, for three consecutive work days. Pollutants examined were total diesel PM_2.5_ (DEP_2.5_) and PAH content. 8-OHdG levels were significantly higher for inspectors vs. controls on days 2 and 3. Increased PAH concentrations in DEP_2.5_ were significantly associated with increased 8-OHdG in the exposed group even after adjusting for factors such as smoking.

Lai et al. [81] studied 47 female toll workers in Taiwan, matched with female office workers as controls; pollution concentrations were not measured. Non-smoking toll workers had significantly higher 8-OHdG levels than did non-smoking controls; increases in 8-OHdG were almost 5 times higher per 1000 trucks/buses than per 1,000 cars (comparing car toll lanes with truck/bus lanes), although these results did not reach statistical significance.

Sauvain et al. [78] studied 32 Swiss bus maintenance workers. PM species examined included PM_4_, OC, EC, metals (Fe, Mn, Cu), and PAHs. Urinary 8-OHdG concentrations increased within each work shift, and between two consecutive work days. Among non-smokers, increased 8-OHdG concentrations were associated with increased levels of OC and Cu. While secondary OC associations were not found using a different measure of oxidative stress (blood levels of Cu, Zn-SOD) in the Delfino et al. studies [74,75], primary OC was significantly associated with oxidative stress biomarkers, suggesting that the OC associations in Sauvain et al. reflect mainly fresh (i.e., primary) diesel emissions.

Sorensen et al. [82] used personal monitors to determine levels of PM_2.5_ and six metals (V, Cr, Pt, Ni, Cu, Fe) in PM_2.5_ fractions to which 49 young, non-smoking students in Copenhagen were exposed. No associations were found with 8-oxodG in urine, but significant positive associations were found for V and Cr with 8-oxodG in lymphocytes.

Kim et al. [76] examined association of urinary 8-OHdG with six PM_2.5_ metal species (V, Cr, Mn, Ni, Cu, Pb) among boilermakers repairing oil-fired boilers in the Boston, MA area. Post-work shift levels of 8-OHdG were significantly higher than pre-work shift levels. Increased 8-OHdG was associated with increases in total PM_2.5_ and V, Mn, Ni, Pb, but not Cu or Cr. PM levels were considerably higher than ambient; for example, the concentration of V was 1.23 μg/m^3^, contrasted with contemporary Boston ambient V levels of 3 to 5 nanograms/m^3^. It is interesting that Cu, but not Mn, was associated with oxidative stress in Sauvain et al. [78], whereas opposite associations were observed for each in Kim et al.

Allen et al. [83] studied 10 adults, aged 18-49, with metabolic syndrome but no history of ongoing medical care for heart disease, hypertension, asthma, diabetes, or similar chronic conditions. Subjects were exposed for two hours either to diesel exhaust (200 μg/m^3^ PM_2.5_) or filtered air. No significant associations were found between urinary 8-OHdG and diesel exhaust exposure. These findings contrast with studies noting increased urinary 8-OHdG with work shift exposure to diesel exhaust in Taiwan [80] and Switzerland [78], or to heavy vehicular traffic in Taiwan [81] and Beijing [79]. The lack of significant findings of oxidative stress in Allen et al. [83] might have to do with shorter exposure times, with differences in makeup of pollution, with differences in levels of activity, i.e., workers presumably were more physically active during exposure than passively exposed subjects in Allen et al., as suggested by the authors, or to other as yet unknown reasons.

Jacobs et al. [84] chose a unique method of assessing pollution exposure, i.e., by measuring the area occupied by carbon in respiratory tract macrophages, in 79 Belgian non-smoking adult diabetics. The authors also examined distance between subject residence and major roads by geocoding. The biomarker measured was oxidized LDL in plasma, a risk factor for atherosclerosis. They found that both increased carbon loading and decreased distance from major roads were significantly associated with higher levels of oxidized LDL. Similarly, using an animal model, namely atherosclerotic apolipoprotein E knockout mice, Lund et al. [93] found that acute exposure to a mixture of gasoline and diesel engine emissions resulted in increased vascular and plasma markers of oxidative stress and the expression of proatherogenic factors.

Kipen et al. [85] exposed healthy young subjects in New Jersey either to diesel exhaust or to a laboratory-generated PM_2.5_ secondary organic carbon atmosphere (~ 190 μg/m^3^ each), with filtered air control for each exposure. Each pollutant atmosphere caused an increase in white blood cell proteasome activity, and the diesel exhaust caused an increase in red blood cell proteasome activity as well, which the authors suggested to indicate oxidative stress. The Delfino et al. studies [74,75] in Los Angeles did not find oxidative stress associated with secondary organic carbon, but the laboratory-generated SOA atmosphere of Kipen et al. did result in such an association. The SOA generated in the laboratory used vegetative organic gases, while the ambient SOA in Los Angeles presumably is mostly from vehicular-derived organic gases. Whether differences in chemical composition due to the differences in source or differences in methods of assessment of oxidative stress, or whether the higher concentrations in the laboratory SOA compared to ambient levels can explain the disparate findings between the Kipen et al. and Delfino et al. studies, is not clear.

While all of the studies noted above directly examined markers of oxidative stress, the results, however, cannot be neatly summarized for several reasons:

· Several studies, in particular those involving urban emissions, did not measure PM_2.5_ species, and only a few measured PM_2.5_ metals, which have a high potential for inducing oxidative stress [3].

· Studies were done in several countries, with varying levels and likely composition of urban air pollutants.

· Although most of the studies used urinary 8-OHdG as a biomarker of oxidative stress, some studies used other markers, and it isn’t clear *a priori* whether oxidative stress impacts of PM on telomere erosion are equivalent for various markers.

Given these limitations, we offer the following assessment of these studies:

· In studies performed in the U.S., primary vehicular emissions, such as EC/BC and primary OC, were associated with increases in biomarkers of oxidative stress, but secondary organic carbon, presumably mostly of vehicular origin, was not.

· In studies performed in Europe and Asia, oxidative stress measures were associated with greater exposure to ambient emissions from roads or at tolls (vs. office worker controls); for those driving in urban vs. suburban/rural areas or living closer to roads; for those with higher levels of carbon in airway macrophages; or for those with greater exposure to emissions found in work locations involving diesels or buses. While exposure to these different atmospheres dominated by vehicular emissions clearly resulted in oxidative stress, little information was generally provided as to which specific components of such atmospheres may have been involved. Only one study examined specific PM_2.5_ species, finding associations with OC and Cu, but not EC, Fe, or Mn in bus maintenance workers.

· Exposures to atmospheres particularly high in PM_2.5_ metals (boilermakers) or to ambient levels of specific metals such as V were associated with oxidative stress. However, only three studies examined metals, and associations were not always with the same metals.

Overall, the studies reviewed in this section suggest that exposure to specific PM sources, namely vehicular, are associated with oxidative stress, but because few of the studies included specific PM species, it isn’t possible as yet to determine which species were most likely responsible for the oxidative stress. Studies in the next section, using a different proxy for oxidative stress, namely change in heart rate variability (HRV), may provide more details on the potential for several PM species to be so associated.

#### Studies providing measures of HRV

Heart rate variability is a reflection of the autonomic nervous system control of cardiac function. Altered HRV has been noted in patients with hypertension and heart failure, and has been linked to increased CVD mortality [92]. Cascio [94] noted that increased cardiac-related deaths and decreased autonomic control of cardiac function can be modulated, at least in part, by oxidative stress. Furthermore, an apparently causal relationship between oxidative stress and changes in various measures of HRV has been found with exposure to pollutants in a number of studies, as discussed below.

Schwartz et al. [95] found significant decreases in the HF component of HRV in people having a deletion for the protein *GSTM1*, part of the cellular defense against oxidative stress, after exposure to PM. For those that did not have the deletion, however, there were no changes in the HF component. Use of statins protected those with this genetic deficiency against oxidative stress associated with exposure to air pollution. Chahine et al. [96] also found that only those people lacking genes protective against oxidative stress, including a different genetic variant than examined by Schwartz et al. [95], experienced reductions in two measures of HRV after PM exposure compared to normal controls. They concluded that oxidative stress was an important pathway for the effects of particles on autonomic nervous function. This is supported by an animal study [97], in which a significant increase in the SDNN component of HRV, which reflects beat-to-beat intervals, and a significant decrease in the ratio of high frequency (HF) to low frequency (LF) components 30 minutes after exposure to urban air particles by instillation was associated with increased oxidative stress in hearts of rats; however, SDNN remained at normal levels when increased oxidative stress was prevented by administration of anti-oxidants prior to exposure (LF/HF was not analyzed in this regard). Finally, Probst-Hensch et al. [98] found that individuals deficient in “oxidant-scavenging” glutathione *S*-transferase (GST) genes had significant HRV alterations, compared to those not so deficient.

Taken together, these studies indicate that changes (mainly reductions) in HRV after exposure to pollutants can reflect induced oxidative stress. Brook et al. [2] noted that “…pathways that reduced endogenous oxidative stress have a protective effect that mitigates reductions in HRV due to ambient PM exposure.” Overall, these studies suggest that associations between oxidative stress and changes in measures of HRV are causative, and that such HRV changes can be considered to be markers of oxidative stress induced by pollutant exposure, in this sense parallel to increases in 8-OHdG found in studies discussed in previous sections. This section reviews studies of HRV changes in people for whom exposure to pollutants, such as BC, was reasonably well characterized, for instance, with use of personal monitors, with ambient monitors which traveled with subjects, with monitors in close proximity to a road to which nearby subject residences were also in close proximity, or by use of wind back- trajectory analysis.

Suh and Zanobetti [72] used both personal monitors and a central pollution monitor to assess exposure to BC, sulfate, and PM_2.5_ in the Atlanta area. The authors found no significant associations with five different measures of HRV when central monitor readings were used. However, risk estimates were 3 to 45 times higher, and BC was significantly associated with changes in all five measures of HRV (Table 2), when personal monitors were used. Thus, for a pollutant such as BC, having large spatial variability across a metropolitan area, use of central monitor readings failed to accurately characterize differences in exposure to BC and understated the extent and significance of HRV changes and, thus, of related oxidative stress, relative to more accurate exposure estimates for BC. No HRV associations were noted for either sulfate (only personal monitor data available) or total PM_2.5_.

Adar et al. [86] used a mobile monitor which followed elderly non-smoking retirees during their activities. Both BC and PM_2.5_ concentrations were sharply higher when subjects were on buses than at other times. HRV changes (5 min and 24 h means) were essentially monotonic with changes in BC and PM_2.5_ concentrations, and were significant in the great majority of cases for both pollutants.

Schwartz et al. [87] examined four different measures of HRV for 1 and 24 h periods. Subjects lived in apartments adjacent to a major Boston roadway; the monitor was also in close proximity to the roadway. BC was consistently associated with changes in all four HRV measures evaluated, while PM_2.5_ was only occasionally associated. The authors then used an algorithm to remove BC from PM_2.5_ each hour, and termed the remainder “secondary PM,” which contained regional sulfate and SOC but not BC. They found no associations with HRV changes and secondary PM. Furthermore, SDNN declined monotonically with increasing PM_2.5_ from the lowest PM_2.5_ level to about 18 μg/m^3^. At these relatively low levels, the investigators noted that PM_2.5_ and BC were highly correlated. However, from about 20 μg/m^3^ to the highest PM_2.5_ levels (about 50 μg/m^3^), the relationship between SDNN and PM_2.5_ was flat, i.e., there were no changes in the SDNN measure of HRV. At these higher PM_2.5_ concentrations, PM_2.5_ was no longer well correlated with BC.

Ebelt et al. [88] examined HRV changes in 16 non-smoking patients with COPD in Vancouver, Canada. Both ambient and personal monitoring were used to obtain measures of PM_2.5_; personal monitoring of sulfate was also performed. Non-sulfate PM_2.5_ was determined by various algorithms which effectively subtracted personal sulfate exposure from PM_2.5_ measures. Of the different measures of PM_2.5_, only non-sulfate urban PM_2.5_ was associated with changes in HRV (SDNN, R-MSSD). These results are the obverse of those of Schwartz et al. [87], in that Ebelt et al. subtracted sulfur to determine urban non-sulfate, which would include BC. However, in both studies, ambient PM reflecting BC or urban air, but not sulfate or regional air, was associated with altered HRV.

Creason et al. [89] examined the relationship between the HF and LF measures of HRV with PM_2.5_ concentrations over 24 consecutive days in 56 elderly, non-smoking men. Sulfate was measured, and reported to average 14% of PM_2.5_ concentrations, but was not reported separately from PM_2.5_. The authors found a “U” shaped relationship between changes in HRV measures and PM_2.5_; HRV changes were most pronounced near the midpoint of PM_2.5_ concentrations, and HRV was most normal at the lowest and highest PM_2.5_ concentrations. When a two day air mass, containing the highest and 3^rd^ highest PM_2.5_ concentrations was removed from the analysis, for the remaining 22 days a monotonic reduction in HF was observed with increasing PM_2.5_, similar to the reduction in SDNN observed with increasing BC by both Adar et al. [86] and Schwartz et al. [87]. The authors used back-trajectory analysis to show that the two day air mass, having PM_2.5_ concentrations of approximately 53 and 37 μg/m^3^, came from rural Pennsylvania, whereas on the other 22 days, the authors suggested that urban or industrial PM_2.5_ sources were present. The lack of association with PM_2.5_ on days dominated by rural air masses parallels the findings of Schwartz et al. [87] that PM_2.5_ in secondary air masses was not associated with changes in HRV, even though PM_2.5_ levels were highest on such days.

Park et al. [90] studied 497 subjects using back trajectory analysis to create six different source directions to assess exposure. Of primary interest are the four directions which had very similar BC, PM_2.5_, and sulfate concentrations (e.g., PM_2.5_ between 14.1 and 15.1 μg/m^3^; BC between 0.99 and 1.1 μg/m^3^; and sulfate between 3.8 and 4.2 μg/m^3^), substantially higher than the remaining two directions (N and NW). These four directions were:

· SW (air mass passed over Philadelphia and New York before following the I-95 corridor toward Boston).

· S (air mass originated in the Carolinas and entered Massachusetts from the ocean to the S).

· W (air mass passed over northern Ohio and middle New York state, mostly north of Pennsylvania).

· Local (slow moving air masses, relatively stagnant over Boston).

On days when air masses were less urban (S and W), there were no HRV changes associated with any emissions. When air masses arrived in Boston from the SW, BC was significantly associated with changes in three of four HRV measures; no other PM_2.5_ species were associated with such changes. When the air was slower moving and more local, PM_2.5_, sulfate, and BC were all associated with HRV changes, although BC was associated more consistently.

For two other wind trajectories (N and NW), levels of all pollutants were low relative to the other four directions. An association with PM_2.5_ was observed for the N wind trajectory, and one for sulfate was seen for the NW wind trajectory. The authors suggested that the effect apparently seen for sulfate in this case, despite low sulfate levels, may actually be due to the presence of transition metals. Lippmann et al. [99] found significant reductions in HRV in mice exposed to ambient air in upstate New York only when the wind trajectory was from the NW, passing over a Sudbury, Ontario nickel smelter.

Two studies that did not monitor PM_2.5_ species are included in this section because they demonstrate that HRV changes can be abolished using a face mask that filtered out urban particles, suggesting that the cause of HRV reductions may be traffic-related PM as opposed to associated gaseous vehicular emissions. Langrish et al. [91] recruited 15 young, non-smokers in Beijing for a cross-over study. Subjects walked a predetermined course on separate occasions, either with or without a face mask. Personal PM_2.5_ monitors were used. A significant decrease in 24-h SDNN was observed when subjects did not use face masks, suggesting production of oxidative stress. Significant reductions in LF were also observed, but interpretation of this effect wasn’t straightforward, since LF reflects tone of the sympathetic nervous system and it might have been affected to some extent by simply wearing a mask. Beijing PM_2.5_ levels when face masks were used were 140 μg/m^3^ vs. 86 μg/m^3^ when masks were not used. In another study, Langrish et al. [92] made similar findings, using subjects with coronary artery disease (average age 62). In addition, they also studied other CVD endpoints, such as ST-segment depression and arterial blood pressure, and found that use of the face mask reduced both. Using electron paramagnetic resonance, the authors also established that ambient PM in Beijing has very high oxidative stress potential.

The results of the studies reviewed in this section may be summarized as follows:

· When there were reasonably accurate subject exposure measures, BC was consistently significantly associated with alterations in various measures of HRV. Among these studies, those able to distinguish BC from secondary air masses without BC, rural air masses from those with industrial or urban pollution, urban PM without sulfate vs. those containing sulfate, or personally monitored BC from personally monitored sulfate, HRV measures were influenced by BC or urban or industrial air, but not sulfate, regional air masses containing low levels of BC, or rural air masses, even though high in total PM_2.5_. In these studies, PM_2.5_ was consistently associated with HRV changes when it was highly correlated with BC, but less so otherwise.

· Although metals were not monitored in any of the HRV studies, the sulfate finding in Park et al. [90] for the air mass with low PM and low sulfate which passed near the Sudbury nickel smelter is suggestive of the Ni association found by Lippmann et al. [99], i.e., HRV was altered in a mouse model only when the wind trajectory was such that the air mass passed near the nickel smelter.

· Those pollutants that were associated with alteration in HRV were also associated with production of oxidative stress which, as noted, can mediate HRV changes.

## Conclusions

The development of CVD is due to both genetic and environmental factors. However, at an individual level, both susceptibility to and the chronological age at onset of CVD varies between subjects who may have similar classical risk factors. A portion of this interindividual difference likely reflects differences in biological aging. Furthermore, there is evidence that oxidative stress may be a pathogenetic mechanism/risk factor that underlies both such aging and the development of CVD, since oxidative stress has been shown to be related to the initiation and progression of CVD.

Shortened telomere length is a marker of biological age and a key process related to development of age-related pathology. Telomere shortening itself appears to be a cause of increased rates of morbidity and mortality, both directly as tissue function normally gradually declines, and indirectly as accumulation of senescent cells accelerate development of pathologies.

Studies discussed in this paper have shown that oxidative stress accelerates telomere erosion, and that strategies to reduce oxidative stress can help maintain telomere length. Furthermore, biological aging appears to be related to the cumulative burden of oxidative stress. Telomeric length seems to be related to the development of CVD in certain population groups, oxidative stress appears to be a consistent risk factor for development of CVD, and mean telomere length can act not only as a marker of this process, but a cause.

Vehicular emissions in various settings have been associated with development of oxidative stress. Based upon a small number of investigations examining metals in PM_2.5,_ such species as Ni, V, Cu, Zn, Mn, and Cr have all been linked to some measure of oxidative stress. Studies reviewed herein also found that BC and primary organic carbon is strongly, and consistently, associated with various measures of oxidative stress when there was accurate subject exposure. Secondary air masses, rural air masses or sulfate when examined in HRV studies with accurate exposure assessment were not associated with oxidative stress in these studies.

Since certain components of air pollution or specific types of pollution have been found to result in oxidative stress, it is conceivable that this mechanism underlies an array of diseases associated with increasing age, in particular CVD, the leading cause of death in the U.S. In this review, we have proposed that oxidative stress caused by exposure to certain air pollutants may be responsible for increased mortality and morbidity and for accelerating a host of CVD states or risk factors, by means of accelerating the erosion of telomere length. If this hypothesis is true, then it is possible that air pollutants which cause oxidative stress might be responsible for the majority of air pollution-related CVD mortality and morbidity.

In sum, there is substantial evidence to believe that vehicular emissions, and specific components of such emissions, shorten lives by accelerating the erosion of telomeres via oxidative stress, thus advancing various cardiovascular diseases. This may constitute a generalized pathway by which oxidative stress may induce much of the morbidity/mortality due to vehicular emissions, as hypothesized by the Health Effects Institute [4], although any role of oxidative stress in telomere erosion was not mentioned in that Report. This proposed pathway may also explain the finding that reduction of a unit of ambient BC was found to extend life 4 to 9 times more than reducing a comparable amount of total PM_2.5_[100]. More analysis is needed to understand the extent to which other PM_2.5_ species may be involved, and also to understand which measures of oxidative stress can most reliably predict accelerated telomere erosion.

### Uncertainties and research recommendations

There are a number of questions and uncertainties that remain to be addressed in relation to the hypothesis proposed in this paper.

Various biomarkers are often used to reflect environmental pollutant exposures and/or as a surrogate measure of a disease process [101,102]. In order to be useful in this regard, the marker must have some predictive validity, but this is often not the case in that while some markers may be generally indicative of oxidative stress, they may have little relationship to disease processes or outcomes [101,103]. Part of the problem may lie with artifacts produced during the sample collection to sample analysis progression, or due to interaction with factors not being assessed [101]. Furthermore, it is not always clear whether specific markers are reflective of acute or chronic exposure to an oxidative stressor. In addition, a biomarker measured in one cell or tissue may not reflect levels and activity in other cells or tissues and, thus, may not be an appropriate surrogate; therefore, these markers cannot be used interchangeably. [104]. While oxidation products of nucleic acids, lipids and proteins may be found in various tissues, our understanding of the relationship between titers in various tissues and disease processes is often unclear [105]. Thus, with regard to the role of oxidative stress in accelerating the rate of telomere shortening, we need to understand if different markers for detecting oxidative stress are essentially equivalent in indicating the extent of such shortening and under what exposure conditions they may or may not be so reflective. In other words, we need a better understanding of which measures of oxidative stress are unambiguously associated with accelerated telomere erosion.

It is interesting to note that cigarette smoke, a known etiological factor for CVD, has been shown to correlate with accelerated shortening of telomeres in some studies but not in others [106]. It has been suggested that since the correlation between telomere length and smoking appears to be small, it is detectable only with large sample sizes and many studies may have been too small to detect any such association [102,106]. Furthermore, the literature that relates cigarette smoking to biomarkers of oxidative stress also provides conflicting results, and this may also be due to small or selected samples or utilization of ambiguous markers [104,107]. It is conceivable that some inconsistency with markers of oxidative stress, telomere length and exposure to air pollutants, as noted herein, may be due to these factors as well.

There needs to be a fuller understanding of which PM_2.5_ species cause oxidative stress. It is crucial to have more human panel studies with accurate exposure and which include metals among the PM_2.5_ species examined. Another way to test which PM_2.5_ species may cause adverse effects is to perform more crossover studies with face masks in different settings, so as to rule out certain PM components of such air masses as causes of oxidative stress. This will help in setting regulatory policy to protect public health if only particles, and not associated gases, continue to be causally related to oxidative stress.

It would be useful to undertake further studies to confirm (or otherwise) that secondary air masses with little vehicular or industrial emissions do not cause oxidative stress. One way to do such tests would be to examine people in areas dominated by rural air masses enriched with secondary organic carbon and sulfate, but far from diesel sources, roadways and industry. Tests for circulating and urinary biomarkers and HRV changes could be conducted in concert with monitoring that would include metal species, some of which can apparently cause adverse health effects hundreds of miles from their source even when present in trace amounts.

The exposure issues noted above would apply even more to studies in Asia and Europe, where close proximity to traffic or to operating diesels away from traffic (e.g., in repair shops or inspection stations) was the metric for exposure, with pollutants such as BC often not monitored. In near-road environments, it will be important to see if components of road dust, often marked by Si, might also cause oxidative stress. Perhaps a three way test could be used: (A) a road with almost no diesels, in a dry area where road dust would accumulate; (B) a road with almost no diesels, in a wetter area having little road dust accumulation, so that particles would mostly be from vehicular emissions but less from road dust; and (C) a road with many diesels. Effects with and without face masks could be compared, at varying distances from each road, in combination with personal pollution monitors. It is possible that face masks might not prevent oxidative stress in every case, and it is possible that oxidative stress may not be present in every case, which would again be useful knowledge for protecting public health.

In spite of results from animal and *in vitro* studies, the ineffectiveness of administered antioxidants in reducing CVD morbidity or mortality in some human clinical and intervention trials has led to a questioning of the importance of oxidative stress in disease pathogenesis [108,109]. For example, a comprehensive study found that standard over-the-counter antioxidants appear not to have “expected” beneficial effects on reducing CVD mortality and morbidity [110]. The Antioxidant Supplementation in Atherosclerosis Prevention [111], a study in which 520 men and women were randomized to receive either Vit. E, slow release Vit. C, or a combination of the two, found that men, but not women, benefitted from the combination therapy in terms of the extent of progression of carotid intima media thickness. Other anti-oxidants, such as NAC (N-acetyl-cysteine), might be more helpful than standard vitamins, but seem to have received less study. NAC acts as an antioxidant by replenishing levels of glutathione (GSH), a crucial anti-oxidant produced by the body, and has a longer biological half-time in the body than do “over-the-counter” anti-oxidants such as vitamin C. Another study found that oxidative stress induced by pollution in nursing home residents in Mexico City was significantly reduced by dietary supplementation of omega-3 fatty acids [112]. Thus, it may be that the mixed results in reducing CVD with antioxidants may be due partly to the types of anti-oxidants customarily used, and/or in part to the lack of specific and sensistive biomarkers by which to assess oxidative stress phenotypes that underly CVD [108]. If the hypothesis that air pollutants shorten telomeres via oxidative stress is substantiated, then discovering how to reduce oxidative stress via inexpensive interventions becomes a high priority.

## Competing interests

TG and RBS declare no competing interests.

## Authors’ contribution

Both authors contributed throughout the report in terms of writing and editing. TG largely contributed discussions of oxidative stress effects of air pollution, RB largely contributed discussions of effects of oxidative stress on telomere length and relationship of CVD to telomere length and oxidative stress. All authors read and approved the final manuscript.

## Disclaimer

This article is dedicated to the memory of Richard Wolf. The opinions expressed herein are those of the authors and do not necessarily reflect the opinion or policy of the United States Department of Energy.
